# Effect of Smoke-Free Legislation on Adult Smoking Behaviour in England in the 18 Months following Implementation

**DOI:** 10.1371/journal.pone.0020933

**Published:** 2011-06-15

**Authors:** John Tayu Lee, Stanton A. Glantz, Christopher Millett

**Affiliations:** 1 Department of Primary Care and Public Health, Imperial College London, London, United Kingdom; 2 Division of Cardiology, Department of Medicine, University of California San Francisco, San Francisco, California, United States of America; 3 Center for Tobacco Control Research and Education, University of California San Francisco, San Francisco, California, United States of America; Yale University School of Medicine, United States of America

## Abstract

**Background:**

Comprehensive smoke-free legislation covering all enclosed public places and workplaces was implemented in England on 1 July 2007. This study examines the impact of this legislation on smoking prevalence, number of cigarettes smoked and location of smoking, controlling for secular trends through the end of 2008.

**Method and Findings:**

Repeat cross sectional survey using nationally representative data from the Health Survey for England (HSE). In total there are 54,333 respondents from 2003–2008. Logit and linear regression models were used to examine the effect of the legislation on smoking prevalence and the number of cigarettes smoked daily among continuing smokers which took the underlying trend into account. Our finding suggest that smoking prevalence (current smoker) decreased from 25% in 2003 to 21% in 2008 (AOR = 0.96 per year, 95% CI = 0.95–0.98, P<0.01) and the mean number of cigarettes consumed daily by smokers decreased from 14.1 in 2003 to 13.1 in 2008 (coefficient for time trend = −0.28±0.06 SE cig/day per year, P<0.01). After adjusting for these trends the introduction of smoke-free legislation was not associated with additional reductions in smoking prevalence (AOR = 1.02, 95% CI = 0.94–1.11, P = 0.596) or daily cigarette use in smokers (0.42±0.28 SE; P = 0.142). The percentage of respondents reporting smoking ‘at work’ and ‘inside pubs or bars’ decreased significantly from 14% to 2% (p<0.001) and from 34% to 2% (p<0.001), respectively, after the legislation. The percentage reporting smoking ‘inside restaurants, cafes, or canteens’ decreased significantly from 9% to 1% (p<0.001) and ‘inside their home’ decreased significantly from 65% to 55% (p<0.01).

**Conclusion:**

There is widespread compliance with the smoke-free legislation in England, which has led to large drops in indoor smoking in all venues, including at home. Declines in smoking prevalence and consumption continued along existing trends; they did not accelerate during the 18 months immediately following implementation.

## Introduction

Comprehensive smoke-free legislation covering all enclosed public places and workplaces was implemented in England on 1 July 2007, following implementation of similar legislation in other parts of the United Kingdom (Scotland in March 2006 and Wales and Northern Ireland in April 2007). The hospitality industry (public bars, clubs and restaurants) was most affected by the legislation because the workplaces of approximately half of employed persons were already smoke-free as a result of voluntary action [1]. The legislation has been well received by the public with 79% in support in 2009 [2], [3] and inspections of premises by enforcement authorities indicate near universal compliance (98–99%) [4]. As has been demonstrated elsewhere, there is accumulating evidence from the UK that this legislation has resulted in substantial population health gain [5]. This includes reduced exposure to second hand smoke, among hospitality workers [6] and the general public [7], [8], and reduced hospital admissions for acute myocardial infarction [9], [10] and childhood asthma [11].

While the primary purpose of the legislation was to reduce exposure to second hand smoke in enclosed public places a secondary objective was to “help people trying to give up smoking by providing supportive smoke-free environments” [12]. This objective is supported by an early systematic review which found that implementation of totally smoke-free workplaces was associated with a 3.8% reduction in smoking prevalence and lower cigarette use in continuing smokers [13]. However, more recent international evidence on the impact of smoke-free legislation on smoking behaviour has produced mixed findings and most studies did not adjust for underlying trends [14], [15], [16], [17], [18]. A recent study of 21 jurisdictions (not including England) that did consider pre-existing secular trends found that comprehensive smokefree laws were associated with acclerations in declines in prevalence in 8 of these jurisdictions and that the laws did not affect the trend in 13 others [19]. Preliminary studies suggest that smoke-free legislation in the UK was associated with a greater number of quit attempts during the first two months after implementation in England [20] and increased sales of nicotine replacement therapy (NRT) and contacts with smoking cessation services in the months prior to implementation in Scotland [21], [22]. However, little is known about how many people were actually successful in quitting smoking or reduced their cigarette use as a result of the legislation. This study aims to examine the impact of this policy on smoking prevalence, volume of cigarettes smoked, and where people smoke in England using data using nationally representative survey data.

## Methods

### Sampling and Data Collection

This study used pooled cross-sectional data from six waves (2003–2008) of the Health Survey for England (HSE). The HSE is an annual survey of people living in private households and is a primary mechanism for monitoring population health in England. The survey is conducted by the National Centre for Social Research and University College London on behalf of the NHS information Centre. The methods of the survey are described in detail elsewhere [23]. Briefly, a two-stage stratified sampling process is employed to obtain an independent, national representative sample each year. The core sample from the general population is boosted by sampling from population groups of interest in some years i.e. persons from ethnic minorities in 2004, older persons in 2005. Interviewers obtain household, socioeconomic and personal details, information on health and illness, and health service use from respondents. Respondents aged above the age of 16 years are then visited separately by a trained nurse. The nurse visit involves anthropometric measurements and collection of saliva samples (which can be used to test for cotinine a stable metabolite of nicotine used to validate self-reported smoking behavior) in some years. All measurements are taken according to survey protocols. This study only looked at respondents from the core sample in each year as we are not able to examine impacts on specific sub groups. We excluded respondents aged under 18 years for our study because: (1) workplaces most affected by this legislation (pubs and clubs) have restricted access to persons under eighteen years of age (2) legislation increasing the age for the legal purchase of cigarettes from 16 to 18 years was introduced in the same year (1 October 2007) (3) children and adolescents have been found to substantially under-report regular smoking in the HSE. Individuals with missing values in any of the variables used in the analysis were also excluded from the study (14.3%). The characteristics of respondents with missing data were not substantially different from HSE respondents in general, suggesting that the data were missing at random. In total there were 54333 observations across from 2003–2008 (12559 in 2003; 5660 in 2004; 6344 in 2005; 11730 in 2006; 5633 in 2007; 12407 in 2008). The number of observations, derived from the core sample of the HSE, was smaller in 2004, 2005 and 2007 because the survey had a boost sample (and a smaller core sample) in those years.

### Study variables

Our main outcome variable was self reported smoking as determined by responses to the question “Do you smoke cigarettes at all nowadays?” (Note that this question will capture both regular daily and occasional nondaily smokers). Secondary outcome measures were volume of cigarettes smoked and location of smoking. Our main predictor variable was the introduction of smoke-free legislation on 1 July 2007. Covariates in our analysis were age, sex, social class and frequency of alcohol use. We categorised age into three groups (18–34, 35–69, 70+ years) and collapsed social class from six (I–professional, II–managerial and technical, IIIN–skilled non-manual, IIIM–skilled manual, IV–partly skilled, V-unskilled) into two occupational groupings (non-manual, manual) for the analyses to increase the power of test. Frequency of alcohol use was categorised into four groups: daily use; use on 3–6 days a week; use on 1–2 days a week; less than weekly use.

### Statistical Methodology

We conducted two separate analyses to examine the effect of the legislation on smoking prevalence and number of cigarettes smoked each day among continuing smokers [24], [25]. The model for smoking prevalence is a binary outcome equation that models *Pr (smoking Status = 1)* using a logit model. To model consumption among continuing smokers we used linear regression which models *E,number of cigarettes a day-smoking status = 1*, where E is the expected number of cigarettes consumed a day if the person is a smoker. The main predictor variable is the implementation of smoke-free legislation in England. We include year, with years other than 2007 (the year the law took effect) set to mid-year, i.e., we set year to 2006.5 for all data collected in 2006. For 2007, we set year to 2007.25 for data collected before the law took effect on 1 July 2007 and 2007.75 for the second half of 2007. We then subtracted 2007.5 from all years, so that the date the law took effect became year 0. Presence of the smoke-free law coded as 1 for interviews conducted after the law was introduced on 1 July 2007.

We also investigated whether there was a “hardening effect” [26], [27], i.e. fewer, high consumption smokers, by regressing cigarettes consumed a day per smoker against prevalence with a variable for the smoke-free legislation and all the other covariates used in this study.

Covariates included age group, gender, occupational status and frequency of alcohol use as covariates. Mulicollinearity diagnostics (VIF) were all less than 5, indicating that the assumption of reasonable independence among predictor variables was met. Adjusted odds ratios were reported, heteroscedasticity-consistent standard errors were used for hypothesis testing. In addition, this study also examined the differential effect of the smoking policy on different subgroup by testing interactions terms between smokefree legislation and the above covariates. Because many interaction terms introduced collinearity into the models we also conducted subgroup analysis.

We also examined whether smoking prevalence and number of cigarettes smoked decreased in the six months leading up to the legislation (Jan–June 2007), as has been suggested in some studies [20], [21]. For this analysis, we set the law dummy variable to 1 at 1 Jan 2007 instead of 1 July 2007.

We used multivariate logistic regression model, adjusting for age, gender, socio-economic status and alcohol consumption to examine the impact of the legislation on location of smoking among smokers by comparing the six months in 2007 before and after the legislation went into effect.

Our sample size has 80% power to detect a 5% relative reduction (1% absolute reduction) in smoking prevalence due to the legislation i.e. over and above the underlying trend at the 5% level of significance.

We did not use weighting in our analysis because previous work suggests doing so only has a very small bearing on study results [28]. All statistical analyses were performance using STATA 11.

## Results

The response rate for the Health Survey for England was 73%, 72%, 64%, 61%, 66% and 64% in the years from 2003 to 2008.

### Impact of the legislation on smoking prevalence and number of cigarettes smoked

**Figure 1:**
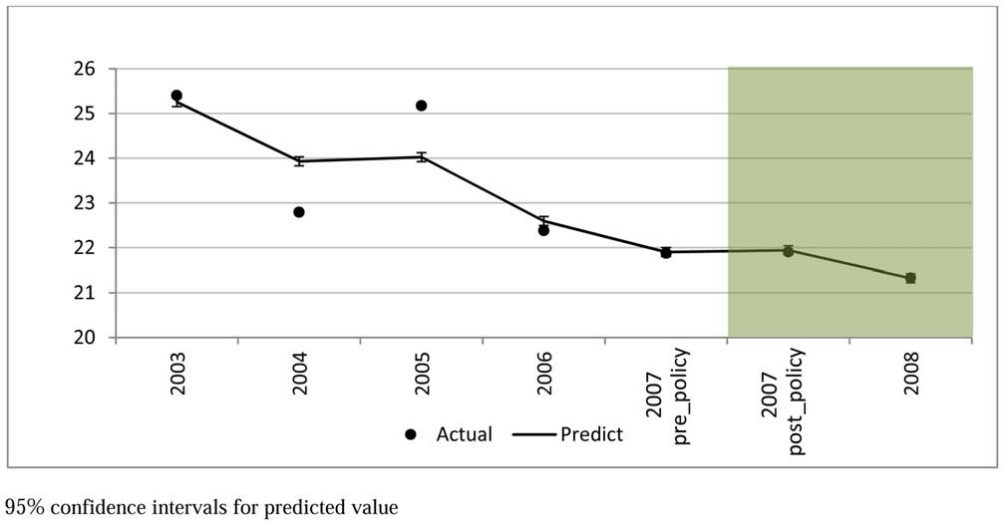
Smoking Prevalence (2003–2008 Mean; 95% CI).

**Figure 2:**
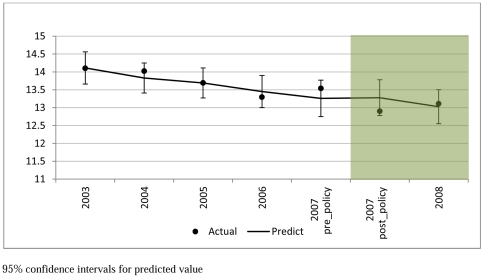
Daily Cigarette Consumption in Smokers (2003–2008 Mean; 95% CI).

Figures 1 and 2 present the smoking prevalence and number of cigarettes smoked per day by smokers in the study period (2003–2008). Smoking prevalence decreased from 25% in 2003 to 21% in 2008. The mean number of cigarettes consumed daily by smokers decreased from 14.1 in 2003 to 13.1 in 2008.

The effect of the smoke-free legislation on smoking prevalence and number of cigarettes smoked among continuing smokers are shown in Table 1. There was a statistically significant time trend with smoking prevalence falling over time (AOR = 0.96 per year, 95% CI = 0.95–0.98, P<0.001) and number of cigarettes smoked (−0.28 cigarettes/day per year, SE = 0.06, P<0.001). The implementation of smoke-free legislation was not associated with a statistically significantly change in the trend in smoking prevalence (AOR = 1.02, 95% CI = 0.94–1.11, P = 0.596) or number of cigarettes smoked per day (0.42, SE = 0.28, P = 0.142). Our investigation of whether there was a “hardening effect” found no significant effect of prevalence (−0.03 per prevalence rate, P = 0.790) and the smoking law (0.433, P = 0.135) on the consumption per continuing smoker.

**Table 1:** Impact of legislation on smoking status.

	Whether smoke or not			Number of Cigarettes per day		
	AOR	P-value	VIF	Coefficient	P-value	VIF
Year	0.96 (0.95, 0.98)	0.0005	2.94	−0.28 (−0.40, −0.16)	0.0005	2.41
Smoke-free legislation	1.02 (0.94, 1.11)	0.596	2.94	0.42 (−0.14, 0.98)	0.142	2.41
Age (18–34 reference)						
35–69years	0.55 (0.52, 0.58)	0.0005	1.48	3.02 (2.71, 3.32)	0.0005	1.18
70+years	0.17 (0.16, 0.19)	0.0005	1.54	−0.38 (−0.98, 0.23)	0.225	1.16
Female	1.07 (1.03, 1.12)	0.002	1.07	−1.43 (−1.73, −1.12)	0.0005	1.10
Manual Occupation	2.19 (2.10, 2.29)	0.0005	1.08	2.00 (1.70, 2.30)	0.0005	1.08
Alcohol consumption (reference less than weekly)						
1–2 days/wk	0.89 (0.84, 0.93)	0.0005	1.42	−1.24 (−1.59, −0.88)	0.0005	1.40
3–6 days/wk	0.87 (0.82, 0.92)	0.0005	1.41	−1.08 (−1.51, −0.66)	0.0005	1.37
almost every day	1.39 (1.30, 1.48)	0.0005	1.34	1.19 (0.72, 1.66)	0.0005	1.33
Constant	−1.05			11.26		

AOR – adjusted odds ratio.

VIF – variance inflation factor.

After controlling for the time trend and all the other covariates as above, the results suggest that there was no significant additional reduction in smoking prevalence (AOR = 1.00, 95% CI = 0.93–1.07, P = 0.905) or number of cigarettes smoked per day (−0.07, SE = 0.257, P = 0.795) in the six months prior to the legislation being implemented.

Older respondents were less likely to smoke than younger respondents (age 18–34) (AOR = 0.55, 95% CI = 0.52–0.58, P<0.001 for the 35–69 years; AOR = 0.17, 95% CI = 0.16–0.19, P<0.001 for 70+ age group) and females were more likely to smoke than males (AOR 1.07, 95% CI = 1.03–1.12, P<0.001). Respondents in manual occupations were more likely to smoke (AOR = 2.19, 95% CI = 2.10–2.29, P<0.001) and smoked more cigarettes per day (2.00 cigarette per day, SE = 0.152, P<0.001) compared to those in non-manual occupations. Compared to people who drink less than weekly, most frequent drinkers (drink almost every day) were significantly more likely to smoke (AOR = 1.39, 95% CI = 1.30–1.48, P<0.001) and the volume of cigarette smoked (1.19 cigarette per day, SE = 0.241, P<0.001) are more as well. We tested for and did not find any significant interactions between the implementation of smoking policy with time, age, sex, occupation or drinking status and our outcome measures (results not shown).

### Impact of the legislation on where people smoke

Data on where people smoke before (1 Jan–30 June 2007) and after (1 July–31 Dec 2007) the legislation are presented in Table 2. The percentage of respondents who reported that they smoke ‘at work’ and ‘inside pubs or bars’ decreased significantly from 15% to 2% and from 36% to 3% respectively. The percentage reporting smoking ‘inside restaurants, cafes, or canteens’ decreased from 9% to 1%. There was a non-significant reduction in those reporting that they smoke ‘inside shops’ and ‘inside other places’. The percentage of respondents who reported that they smoked “inside their home” decreased significantly from 65% to 55%. Smoking ‘whilst travelling by car’ decreased significantly from 32% to 26%. The percentage reporting that they smoke ‘outside’ increased significantly from 45% before the legislation to 63% after.

**Table 2:** Location of smoking before and after the introduction of smoke-free legislation.

Location of smoking	Percentage		AOR for Smoking Law	P-Value
	Before (1 Jan–30 June 2007)	After (1 July–31Dec 2007)		
*At work*	15%	2%	0.12	0.0005
*Inside pubs or bars*	36%	3%	0.04	0.0005
*Inside restaurants, cafes, or canteens*	9%	1%	0.12	0.0005
*Inside shops*	0%	0%	0.50	0.550
*Inside their home*	65%	55%	0.67	0.001
*Inside other peoples home*	16%	13%	0.74	0.065
*Inside other places*	4%	3%	0.81	0.480
*Whilst travelling by car*	32%	26%	0.73	0.015
*Outside*	45%	63%	2.11	0.0005

AOR – adjusted odds ratio; age, gender, socio-economic status, alcohol consumption (same covariates as used in smoking prevalence model) has been controlled in the multivariate logistic regression model.

## Discussion

### Main findings

Our findings indicate an underlying trend of reduced smoking prevalence and decreasing daily cigarette use by smokers in England between 2003 and 2008. After taking this trend into account we found that smoke-free legislation introduced on 1 July 2007 was not associated with a significant acceleration or deceleration in reductions in smoking prevalence or daily cigarette consumption among continuing smokers during the 18 months following implementation of the law. We found no evidence that anticipation of the legislation by smokers resulted in a reduction in prevalence or use in the months prior to implementation. Very few respondents (1–2%) report smoking in workplaces, pubs, cafes or other enclosed public places after the legislation was introduced in keeping with high compliance reported by local enforcement agencies.

### Previous studies

Previous studies suggest increased NRT use, contacts with smoking cessation services and quit attempts leading up to and after the introduction of smoke-free legislation in the UK [20], [21], [22]. Our findings suggest that this increase did not translate into a significant acceleration in reductions in smoking prevalence or daily cigarette consumption in the period prior to and after the introduction of smoke-free legislation in England beyond the established trend. These findings confirm a preliminary analysis undertaken using HSE data which found no significant change in smoking prevalence in respondents interviewed in the six months before and after the legislation was implemented on 1 July 2007 [29].

A previous local study conducted in north-west England 3 months after the law took effect found no significant change in smoking prevalence but found a reduction in the proportion of heavy smokers (≥20 cigarettes per day) [30].

Consistent with previous studies we found that implementation of smoke-free legislation was associated with reductions in smoking in the home and whilst travelling in a car [31], [32], suggesting a shift in social norms around exposing others to second hand smoke in private as well as public places.

### Strengths and limitations

Our study had a number of strengths and limitations. The HSE is a representative national survey and a primary mechanism for monitoring population health in England. By pooling six years of cross sectional data this study is adequately powered to detect a 5% relative deviation from the underlying time trend due to the smoke-free legislation. Having said that, the fact that we only have 18 months of data following implementation of the law does reduce the ability of our study to detect changes in the rate of change over time

The use of cross-sectional data to compare outcomes over time may introduce bias, given that there may be systematic differences in respondents sampled in the different survey years. Our outcome measures were based on self report. We were unable to validate smoking status or daily tobacco use with salivary cotinine measurements as these were not available in all of the study years. A preliminary analysis using 2007 HSE data found significant reductions in cotinine levels among male (316 ng/ml to 276 ng/ml) and female (277 ng/ml to 250 ng/ml) smokers in the six months after the legislation was introduced, consistent with our and others' [23] finding of a continuing decline in daily cigarette consumption among continuing smokers. Previous qualitative research suggests that the English smoke-free legislation may have a differential impact by ethnic group [33]. We were not able to examine this quantitatively in our study because the number of HSE respondents from ethnic minority groups was too small in most years.

### Policy implications

The secondary policy objective of implementing smoke-free legislation in England to “support people trying to give up smoking by providing supportive smoke-free environments” appears not to have resulted accelerating the rate of reduction in smoking prevalence or tobacco use beyond the existing downward secular trend based on the first 18 months after the law was implemented. However, this result may reflect the fact that the majority of employed persons in England worked in smoke-free environments before 2007 and the legislation largely affected the hospitality industry. As the new restrictions thus impacted on relatively infrequent social rather than work activities for most of the population more gradually changes in social norms around smoking behaviour might be anticipated although, as noted earlier, our study suggests that there were modest reductions in smoking in locations not affected by the legislation, i.e., at home and whilst travelling in a car. Another implication of our findings is that there is no evidence of the “hardening” of smokers as prevalence falls. If such hardening were taking place, one would expect that consumption per smoker would increase as prevalence fell. We found just the opposite: as prevalence fell so did consumption per smoker. Globally, most countries have no or very limited smoke-free legislation and enforcement activities are generally weak [34]. Implementation of comprehensive smoke-free legislation as part of Article 8 of the Framework Convention on Tobacco Control may have more discernible impacts of tobacco use in these countries.
